# Supplementary material for: Dynamic transcriptomic profiles of zebrafish gills in response to zinc supplementation
Source: BMC Genomics. 2010 Oct 11;11:553. doi: 10.1186/1471-2164-11-553 (PMC3091702; doi:10.1186/1471-2164-11-553)
Supplement: Additional file 2 — Interactive Direct Interaction Network representing the molecular interactions between zinc, copper, iron, calcium and proteins encoded by transcripts changed by zinc supplementation. Mini web-site containing index.html and hyperlinked pages in subdirectory describing a Direct Interaction Network automatically generated based on curated interactions contained within the proprietary PathwayArchitect database. Ovals represent proteins and the circles symbolize metal ions. Objects are coloured by their abundance in zebrafish at the time-point they were significantly different from the control is a scale from -4 fold (dark green) to +4 fold (dark red). Where significant differences were found at more than one time-point, the colour overlay shows expression at the first instance. Dark blue squares denote 'binding', and light blue squares 'expression'; green squares stand for 'regulation', green diamonds for 'metabolism', and green circles for 'promoter binding'. Arrow heads indicate directionality of the interaction where annotated. All nodes and edges can be further interrogated by selecting the relative area of the image. [file 1471-2164-11-553-S2.zip › PathwayArchitect Zn xs DIN/80308.html]

# PROTEIN: CCNE1

|  |  |
| --- | --- |
| Name | CCNE1 |
| Type | PROTEIN |
| Description | cyclin E1 |
| Note | The protein encoded by this gene belongs to the highly conserved cyclin family, whose members are characterized by a dramatic periodicity in protein abundance through the cell cycle. Cyclins function as regulators of CDK kinases. Different cyclins exhibit distinct expression and degradation patterns which contribute to the temporal coordination of each mitotic event. This cyclin forms a complex with and functions as a regulatory subunit of CDK2, whose activity is required for cell cycle G1/S transition. This protein accumulates at the G1-S phase boundary and is degraded as cells progress through S phase. Overexpression of this gene has been observed in many tumors, which results in chromosome instability, and thus may contribute to tumorigenesis. This protein was found to associate with, and be involved in, the phosphorylation of NPAT protein (nuclear protein mapped to the ATM locus), which participates in cell-cycle regulated histone gene expression and plays a critical role in promoting cell-cycle progression in the absence of pRB. Two alternatively spliced transcript variants of this gene, which encode distinct isoforms, have been described. Two additional splice variants were reported but detailed nucleotide sequence information is not yet available. |
| Alias | CCNE1 |
|  | CCNE |
|  | Ccne1 |
|  | CYCLE |
|  | AW538188 |
|  | cyclin Es |
|  | cyclin Et |
|  | cyclin E1 |
|  | G1/S-specific cyclin E1 |
|  | Ccne |


---

|  |  |
| --- | --- |
| GO Component | cyclin-dependent protein kinase holoenzyme complex |
|  | nucleus |


---

|  |  |
| --- | --- |
| GO ID | GO:0005634 |
|  | GO:0000074 |
|  | GO:0016301 |
|  | GO:0006270 |
|  | GO:0016538 |
|  | GO:0000910 |
|  | GO:0006468 |
|  | GO:0016049 |
|  | GO:0030521 |
|  | GO:0045893 |
|  | GO:0000307 |
|  | GO:0003713 |
|  | GO:0005515 |
|  | GO:0050681 |
|  | GO:0051301 |
|  | GO:0000082 |
|  | GO:0007049 |


---

|  |  |
| --- | --- |
| MIM | MIM:123837 |


---

|  |  |
| --- | --- |
| Connectivity | 813 |


---

|  |  |
| --- | --- |
| Entrez ID | 12447 |
|  | 25729 |
|  | 898 |


---

|  |  |
| --- | --- |
| Agilent ID | A\_51\_P408946 |
|  | A\_53\_P136090 |
|  | A\_14\_P130291 |
|  | A\_14\_P125491 |
|  | A\_42\_P518254 |
|  | A\_52\_P796682 |
|  | A\_44\_P182412 |
|  | A\_14\_P123495 |
|  | A\_23\_P209200 |


---

|  |  |
| --- | --- |
| Cellular Localization | Nucleus |
|  | Organelle |
|  | Cell |


---

|  |  |
| --- | --- |
| DbXref | Reactome##172757##Cell Cycle Checkpoints##http://www.reactome.org/cgi-bin/eventbrowser?DB=gk\_current&ID=172757 |
|  | Reactome##172742##Cell Cycle (Mitotic)##http://www.reactome.org/cgi-bin/eventbrowser?DB=gk\_current&ID=172742 |
|  | Reactome##69278##Cell Cycle (Mitotic)##http://www.reactome.org/cgi-bin/eventbrowser?DB=gk\_current&ID=69278 |
|  | Reactome##69620##Cell Cycle Checkpoints##http://www.reactome.org/cgi-bin/eventbrowser?DB=gk\_current&ID=69620 |
|  | KEGG pathway##04110##Cell cycle##http://www.genome.jp/dbget-bin/show\_pathway?mmu04110+12447 |
|  | KEGG pathway##04110##Cell cycle##http://www.genome.jp/dbget-bin/show\_pathway?hsa04110+898 |


---

|  |  |
| --- | --- |
| Pathway | P53 Signaling |
|  | Zn xs inventory |
|  | Zn xs DIN |


---

|  |  |
| --- | --- |
| GO Process | cell growth |
|  | G1/S transition of mitotic cell cycle |
|  | DNA replication initiation |
|  | positive regulation of transcription, DNA-dependent |
|  | protein amino acid phosphorylation |
|  | cell cycle |
|  | androgen receptor signaling pathway |
|  | cell division |
|  | regulation of progression through cell cycle |
|  | cytokinesis |


---

|  |  |
| --- | --- |
| UniGene | Rn.15455 |
|  | Mm.16110 |
|  | Hs.244723 |


---

|  |  |
| --- | --- |
| Affymetrix Probeset ID | 103034\_at |
|  | 1416492\_at |
|  | 1441910\_x\_at |
|  | 169903\_r\_at |
|  | 171290\_r\_at |
|  | 2021\_s\_at |
|  | 213523\_at |
|  | 242105\_at |
|  | 41060\_at |
|  | 139352\_f\_at |
|  | Hs.244723.0.A1\_3p\_at |
|  | Hs.9700.0.S1\_3p\_at |
|  | M74093\_at |
|  | X75888\_s\_at |
|  | X95406\_at |
|  | 1373530\_at |
|  | D14015\_at |
|  | D14015\_g\_at |
|  | rc\_AI177336\_s\_at |
|  | RC\_T54121\_s\_at |
|  | TC16950\_at |
|  | TC16950\_g\_at |


---

|  |  |
| --- | --- |
| GO Function | transcription coactivator activity |
|  | protein binding |
|  | cyclin-dependent protein kinase regulator activity |
|  | kinase activity |
|  | androgen receptor binding |


---

|  |  |
| --- | --- |
| Nucleotide | BC003499 |
|  | NM\_057182 |
|  | X95406 |
|  | M73812 |
|  | AK178079 |
|  | U40787 |
|  | BC084588 |
|  | L48996 |
|  | AU137789 |
|  | M74093 |
|  | D14015 |
|  | AK177216 |
|  | BC062152 |
|  | BE645164 |
|  | AK165658 |
|  | XM\_574426 |
|  | NM\_007633 |
|  | X75888 |
|  | NM\_001238 |
|  | BC035498 |
|  | AK163046 |
|  | U40788 |
|  | AK089950 |
|  | AF518727 |


---

|  |  |
| --- | --- |
| Protein | BAA03116 |
|  | P24864 |
|  | Q61457 |
|  | AAH35498 |
|  | NP\_031659 |
|  | AAM54043 |
|  | XP\_574426 |
|  | NP\_476530 |
|  | AAA83269 |
|  | NP\_001229 |
|  | CAA53482 |
|  | CAA64687 |
|  | P39949 |
|  | CAA64688 |


---

|  |  |
| --- | --- |
| Organism | Mammal |


---

|  |  |
| --- | --- |
| Location | chromosome 19, 19q12 (Homo sapiens) |
|  | chromosome 1, 1q22 (Rattus norvegicus) |
|  | chromosome 7, 7 16.0 cM, 7 B2 (Mus musculus) |
|  | 7 16.0 cM (Mus musculus) |


---

|  |  |
| --- | --- |
